# Impact of the 2013 Floods on the Incidence of Malaria in Almanagil Locality, Gezira State, Sudan

**DOI:** 10.1371/currents.dis.8267b8917b47bc12ff3a712fe4589fe1

**Published:** 2018-10-08

**Authors:** Yasir Elfatih Abdelrahim Elsanousi, Abbas Suleiman Elmahi, Irene Pereira, Michel Debacker

**Affiliations:** Alsabeel Charitable Health Center (SCHC), Division of Medical & Health Services, Omdurman, Sudan; Department of Preventive Medicine, Ministry of Health, Gezira State, Wadmedani, Sudan; Research Group on Emergency and Disaster Medicine, Vrije Universiteit Brussel, Brussels, Belgium; Research Group on Emergency and Disaster Medicine, Vrije Universiteit Brussel, Brussels, Belgium. Department of Emergency Medicine, Universitair Ziekenhuis Brussel, Belgium

## Abstract

Background: Heavy rain hit Sudan in August 2013 with subsequent flash floods in different parts of the country. This study investigated the impact of the flooding on incidence of malaria in Almanagil Locality in central Sudan.

Methods: This observational retrospective study compared malaria data sets during rainfall seasons in the Almanagil Locality in the year of flooding (2013) with those of corresponding rainfall seasons of previous two non-flood years (2011 and 2012).

Results: A marked increase of new malaria cases and incidence rate was observed in the 13 sentinel malaria notification sites in the locality  (IR increased from 6.09 per 100,000 person­days in 2011 [95 % CI: 5.93-6.26] and 6.48 in 2012 [95 % CI: 6.31-6.65] to 8.24 in 2013 [95 % CI: 8.05-8.43] ; P< 0.0001), with a peaking of the incidence rate in the under-5-years age group (IR for this age group jumped from 9.80 per 100,000 person­days in 2011 [95 % CI: 9.29­10.32] and 10.00 in 2012 [95 % CI: 9.52­10.49] to 15.02 in 2013 [95 % CI: 14.41­15.64]). A noticeable increase in the slide positivity rate (P< 0.0001) was observed in the 12-week period of 2013 (SPR = 20.86% [95 % CI: 20.40 ­21.32%]) compared with the same periods in 2011 (SPR = 8.72% [95 % CI: 8.36 ­9.08%]) and 2012 (SPR = 12.62% [95 % CI: 12.24 ­13.01%]), with a more marked rise of the SPR in the under-5-year age group. Hospital data showed increase in both the inpatient and outpatient incidence proportions in the study period of 2013 compared to those of the years 2011 and 2012. Hospital OPD incidence proportion in 2013 was 19.7% (95% CI: 19.24­20.18%) compared to 12.85% (95% CI: 12.48­13.23%) in 2011, and 12.16% (95% CI: 11.82­12.51%) in 2012. The < 5 year old groups were responsible for the overall rise in the proportion of malaria cases in 2013 , particularly the < 1 year old group which more than doubled in the 2013 period compared to both 2011 and 2012 periods (Age­specific proportion of the outpatient malaria cases of the < 1 year old group in 2013 was19.5% [95% CI: 18.5­20.6%]  compared to 7.7% [95% CI: 6.9­8.6%] in 2011 and 8.1% [95% CI: 7.3­8.9%] in 2012. Incidence proportion of severe malaria cases (inpatients) increased to 22.5 % (95 % CI: 21.5 to 23.6 %) in the study period of 2013 compared to 19.8 % (95 % CI: 18.6 to 21.0 %) in 2011 and 18.4 % (95 % CI: 17.4 to 19.5) in 2012. The increase in the proportion of severe malaria cases was mainly due to a higher proportion of children < 5 years of age and especially to a higher proportion of children < 1 year of age.

Conclusion: The study revealed a significant increase in the incidence rate of malaria in Almanagil Locality following the flash flood of August 2013. The flooding had the highest impact on the malaria incidence of the under-5-years age group, and particularly of the under-1-year age group.

Keywords: Flood, Flooding, Malaria, Disaster, Sudan, Gezira, Almanagil

## Background

Besides the rise in global temperature, climate change also results in an increased frequency of extreme weather events such as floods and droughts 1^, ^2^, ^3 . Floods are the most common natural disasters globally and have led to extensive morbidity and mortality throughout the world especially in low-resource countries 4^, ^5^, ^6 . Impacts of floods on the health of populations depend not just on the magnitude of the flood, but also on geographic, socio-economic and human factors including the vulnerability and adaptive capacity of the population affected 6^, ^7^, ^8 . Climate change, resulting in rising temperatures, changing precipitation patterns and increase in extreme weather events, can affect infectious disease outbreaks by altering vector population, density and survival rates, and pathogen reproduction and development rate 2^, ^9^, ^10 . This in turn will influence human exposure to bites from infected vectors 8 .

Malaria has been identified as one of the diseases most sensitive to climatic factors 10^, ^11^, ^12 . Conflicting results exist on the impact of extreme weather events on malaria transmission 12^, ^13^, ^14^, ^15 . Malaria epidemics attributed to prolonged precipitation and flooding have been reported in endemic areas world-wide 10^, ^12^, ^13^, ^15^, ^16^, ^17^, ^18^, ^19^, ^20^, ^21 . However, other studies showed a decrease in the malaria burden or did not provide evidence in support of causal relationship between malaria transmission and heavy rainfall or flooding 22^, ^23^, ^24^, ^25 . Although the two opinions seem to be contradictory, both viewpoints can be true. Heavy precipitations and floods may initially decrease vector populations by eliminating existing mosquito-breeding sites and, hence, lower malaria transmission 11^, ^12^, ^13^, ^21^, ^26. However, as the heavy rainfall stops and floods waters gradually recede, stagnant pools are created, providing ideal habitats for mosquitoes and resulting in an increase in the vector population and an upsurge of malaria transmission in the following weeks 10^, ^11^, ^19^, ^20^, ^21. Moreover, these differences in the impact of heavy rainfall and flooding may also be the result of the specific geographic setting of the affected area such as topography, distance to water bodies, altitude, climate and land type 12^, ^14^, ^15 . The lag time, which may vary by geographic and climatic conditions, is usually around 4 to 8 weeks between the flooding and the onset of a malaria outbreak 9^, ^16^, ^18^, ^26^, ^27^, ^28^, ^29 .

Malaria is considered a leading cause of mortality and morbidity around the world, with a global 2013 estimate of 198 million cases and about 584000 deaths. Africa accounts for an estimated 90 % of all deaths and children aged under five represented 78 % of all deaths 30 . Malaria in Sudan is endemic 31 and continues to be a major public health problem. The whole population is at varying degrees of risk. Climate models estimate that 75 % of the population of Sudan are at risk of endemic malaria and 25 % are at risk of epidemic malaria 29 . *Plasmodium falciparum* is the main parasite and *Anopheles arabiensis* is the primary vector 32 . A National Malaria Control Program (NMCP) has been established under the Sudan Federal Ministry of Health (FMOH). The country endorsed the international Roll Back Malaria initiative in 1998 emphasizing efforts towards more attention on early detection, prompt treatment and various prevention measures 33 . Collaboration of the NMCP with the United Nations Development Program (UNDP), the World Health Organization (WHO) and national Non-Governmental Organizations to fight malaria in Sudan, resulted in reducing the number of malaria cases from more than four millions in 2000 to less than one million in 2010, and 75% reduction in mortality due to malaria between 2001 and 2010 34^, ^35 .

According to the WHO criteria for risk of malaria transmission and the 2012 Malaria Indicators Survey (MIS), Gezira State is considered hypo­endemic 36 , entailing that the state is more liable to malaria outbreaks. Indoor ­household residual spraying (IRS) and distribution of insecticide treated nets (ITNs) were implemented in Gezira State. The 2012 MIS showed that approximately 95% of the population was protected by IRS, but only 34% of the households owned at least one ITN and only 5% of the households members slept under an ITN 36 .

In 2013, the worst flash floods in 25 years hit Sudan 37 . Continual heavy rains from early August 2013 and consequent flash floods caused extensive damage and loss of life in 15 states. Reports from the Humanitarian Aid Commission estimated 499,900 people countrywide were affected by the heavy rainfall and floods across Sudan since the onset of the events in early August 2013. Assessments have shown that the floods destroyed or damaged over 85,385 houses in the states resulting in the displacement of a large part of the affected population, disrupting the healthcare system, the provision of drinking water and access to sanitation 38 . Gezira State was one of the most affected states: 52,975 affected people, 5,946 houses destroyed and 5198 houses damaged 39^, ^40 . There were concerns about epidemics of communicable diseases including vector-borne diseases such as malaria 41 .

The objective of the study was to estimate the malaria incidence attributable to the 2013 flood in Almanagil Locality, Gezira State in central Sudan.

## Methods

**Study Area**

Gezira State is located in the east-central region of Sudan, is crossed by the Blue Nile and is irrigated by two canals of the Gezira and Managil agriculture schemes. Almanagil Locality was in 2013 one of the 8 localities that constituted the Gezira State and was situated in the south western part of the state. Almanagil Town, the capital of the locality, is 62 km away from Wadmedani, the capital of Gezira State, and 156 km from Khartoum, the capital of Sudan (Figure 1).

**The location of Almanagil Locality in Gezira state in Sudan in 2013.**
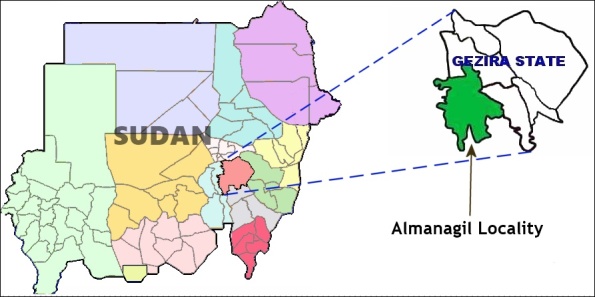

The climate is characterized by an average daily temperature of 32° C during summer and 22° C during winter. The rainy season starts in July/August and ends by October, with an estimated annual rainfall of 140 to 225 mm and a relative humidity of 30% to 38% 42 . The estimated population of the locality in 2013 was 1,050,000 persons, living in 6 towns, 416 villages and 356 agricultural labourer settlements and consisted of 90% rural households 43 .

**Malaria case definition**

The malaria case definition remained unchanged throughout the 3 years of study and was based on the national protocol for diagnosis and treatment of malaria: “A malaria case is confirmed by demonstration of asexual forms (trophozoite stage) of the parasite in a thick or thin peripheral blood film or by rapid diagnostic test (RDT) in the presence of fever" 36 .

**Data collection**

The sources for data collection are twofold: the sentinel surveillance system and the routine health management information system operated by the Gezira State Ministry of Health (MOH). Data on confirmed malaria cases was collected from the sentinel malaria notification sites (SMNSs) of Almanagil Locality. The data was extracted from the weekly reports of 13 SMNSs (9 hospitals and 4 health centres) forwarded to the Department of Malaria Control Programme of the Gezira State MOH for the years 2011, 2012 and 2013. The SMNSs are facilities equipped with laboratories and trained clinical and laboratory staff capable of performing microscopy and RDTs for malaria. Hence the malaria incidence rate (IR) and the slide positivity rate (SPR) are calculated from this data.

Hospital data of outpatient malaria cases was collected by the 9 hospitals of Almanagil Locality. Data of inpatient malaria cases was collected by 8 of the 9 hospitals (the ninth hospital was not yet fully operational in 2013 with only outpatient services). The data was extracted from the monthly reports forwarded to the Statistics Department of the Gezira State MOH for the years 2011, 2012 and 2013. The data consisted of the monthly numbers of all out- and inpatient diagnoses, including the confirmed malaria cases.

As heavy rain and subsequent flash flood occurred in the beginning of August and taking into account that the effect of these climatic factors on malaria is not immediate, a four-week lag effect was assumed 11^, ^27^, ^29 . A new generation of infective vectors needs about 30 days to develop: 15 days for the preimaginal development of vector *Anopheles*, 4-7 days for the gonadotropic cycle for parous/nulliparous female mosquitoes and 12 days for the sporogonic cycle for the *Plasmodium falciparum* parasites in the vector mosquitoes 44 . As a result, the study compared data of the months September, October and November (hospital data registered by Gezira State Ministry of Health) and the corresponding 12 weeks from 36th week to 47th week (data registered by 13 SMNSs) of the year of the flooding (2013) with data of the same periods in the two preceding years without flooding (2011 and 2012).

Estimates of the general population were obtained from the Preventive Medicine Department and under 5 years population from the Vaccination Department in Almanagil Locality.

To determine the impact of flooding on malaria burden in Almanagil Locality the following outcomes of interest were selected: (1) the malaria incidence rate expressed as the number of new malaria cases per person-time, (2) the SPR or test positivity rate (TPR) defined as the number of laboratory-confirmed cases (microscopy or RDT) per 100 suspected cases, (3) incidence proportion of uncomplicated malaria cases as the ratio of the number of malaria-related outpatient visits to the total of all-cause outpatient visits, (4) incidence proportion of severe malaria cases measured as the ratio of the number of malaria-related hospitalisations to the total hospital admissions.

**Data analysis**

The confirmed malaria cases and SPR data were extracted from the SMNSs’ reports of the weeks 36 to 47 of each year representing the months September, October and November of each year under study. The registered data was stratified by age (under 5 years of age and above 5 years old) and gender. The confirmed malaria cases were extracted from the hospital reports of the months September, October and November of each year under study. The registered data was available in a stratified form by age groups (< 1 year, 1-4 years, 5-14 years, 15-24 years, 25-44 years and 45 years and older) and gender. As the time interval of the SMNS and hospital reports is not quite the same, i.e. 12 weeks or 84 days versus 3 months or 91 days, the malaria incidence rate is expressed as the number of new cases per 100,000 persons per day.

Data was exported to Microsoft Excel® 2007 spreadsheets and statistical analysis was performed using MedCalc® version 18. P ≤ 0.05 was considered significant for all tests.

**Ethical considerations**

Ethical clearance for the study was given by the Gezira State MOH.

## Results

**Malaria incidence rate**

Analysis of the confirmed malaria cases detected through passive case surveillance in the 13 SMNSs in Almanagil Locality during the 36th to 47th week of the 3 years under study revealed that the malaria incidence rate was highest in 2013 (Figure 2). A marked IR increase to 8.24 per 100,000 person­-days (95% CI: 8.05-8.43) was noticed in the year of the flood in comparison to the two non­-flood years 2011 (IR: 6.09; 95% CI: 5.93-6.26; P < 0.0001) and 2012 (IR: 6.48; 95% CI: 6.31-6.65; P < 0.0001) as shown in Table 1.

**Table 1: Numbers and incidence rates with 95% confidence intervals of new malaria cases recorded in the SMNSs of Almanagil Locality in weeks 36-47 of years 2011-2013.** 

	Year 2011	Year 2012	Year 2013
Population of Almanagil Locality	990,247	1,019,953	1,049,659
Number of new SMNS cases	5,069	5,549	7,262
Incidence rate per 100,000 person-days (95% CI)	6.09 (5.93-6.26)	6.48 (6.31-6.65)	8.24* (8.05-8.43)
SMNS: Sentinel malaria notification site. * P < 0.0001.			

**Malaria incidence rates with 95% confidence intervals recorded in the SMNSs of Almanagil Locality in weeks 36 to 47 of years 2011 to 2013. * P < 0.0001.**
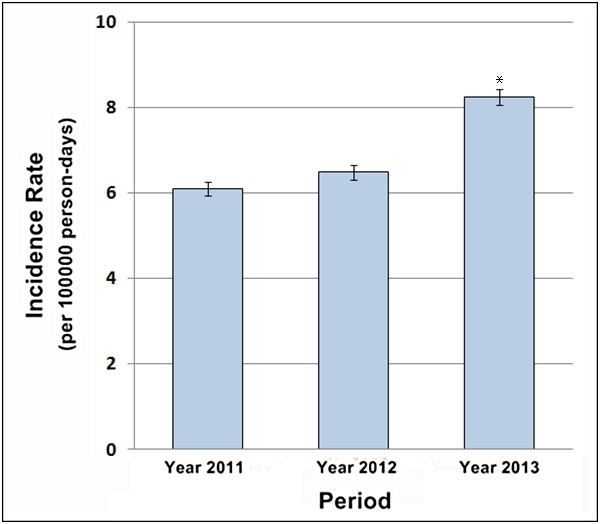

The malaria IR of both age groups (<5 years and > 5 years) increased in the 12-­week period of the flood year (P < 0.0001) compared to the corresponding period in the two non-­flood years. IR for the above­-5-­year age group increased from 5.31 per 100,000 person­-days in 2011 (95% CI: 5.14­-5.49) and 5.64 in 2012 (95% CI: 5.46-­5.82) to 6.80 in 2013 (95% CI: 6.62-­7.00). IR for the under-­5-­year age group jumped from 9.80 per 100,000 person-­days in 2011 (95% CI: 9.29-­10.32) and 10.00 in 2012 (95% CI: 9.52­-10.49) to 15.02 in 2013 (95% CI: 14.41­-15.64) as shown in Table 2 and Figure 3.

**Table 2: Number of new malaria cases and incidence rates by age group with 95 % confidence intervals recorded in the SMNSs of Almanagil Locality in weeks 36 to 47 of years 2011 to 2013.** 

	Year 2011		Year 2012		Year 2013	
Age group	Under 5 years	Above 5 years	Under 5 years	Above 5 years	Under 5 years	Above 5 years
Total number of SMNS cases	11,032	33,645	11,917	37,073	10,603	41,170
Number of malaria cases	1,419	3,650	1,645	3,904	2,307	4,955
Age category proportion from total malaria cases	28.0%	72.0%	29.6%	70.4%	31.8%	68.2%
Population by age group	172,431	817,816	195,840	824,113	182,866	866,793
Age-specific incidence rate of malaria per 100,000 person-days (95% CI)	9.80 (9.29-10.32)	5.31 (5.14-5.49)	10.00 (9.52-10.49)	5.64 (5.46-5.82)	15.02* (14.41-15.64)	6.80* (6.62-7.00)
* P < 0.0001.						

**Age-specific malaria incidence rates with 95% confidence intervals recorded in the SMNSs of Almanagil Locality in weeks 36 to 47 of years 2011 to 2013. * P < 0.0001.**
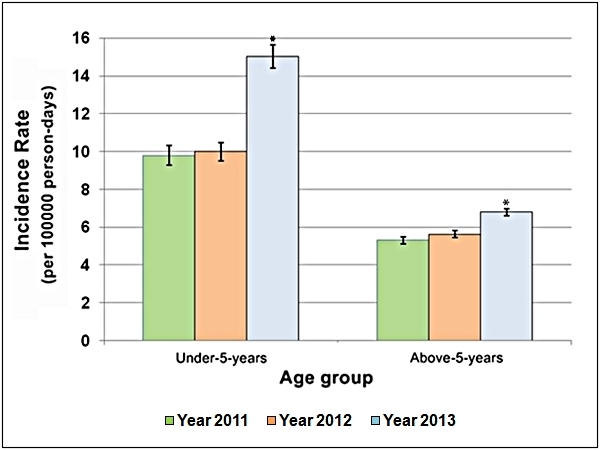

## Slide Positivity Rate

Analysis of the blood smears examined at the SMNSs showed a noticeable increase (P < 0.0001) in the SPR in the 12­-week period of the flood year (SPR = 20.86% [95% CI: 20.40-­21.32%]) in comparison to the corresponding periods in 2011 (SPR = 8.72% [95% CI: 8.36-­9.08%]) and 2012 (SPR = 12.62% [95% CI: 12.24­-13.01%]) as shown in Table 3 and Figure 4.

**Table 3: Number of positive blood smears and SPR with 95 % confidence intervals recorded in the SMNSs of Almanagil Locality in weeks 36 to 47 of years 2011 to 2013.** 

Period	BSE	Number of positives	SPR	95% Confidence interval
All 12 Weeks 2011	23,623	2,059	8.72%	(8.36 -9.08%)
All 12 Weeks 2012	28,903	3,647	12.62%	(12.24 -13.01%)
All 12 Weeks 2013	29,900	6,236	20.86%*	(20.40 -21.32%)
SPR = Slide positivity rate, BSE = Blood smears examined. * P < 0.0001.				

**Slide positivity rate with 95 % confidence intervals recorded in the SMNSs of Almanagil Locality in weeks 36 to 47 of years 2011 to 2013. * P < 0.0001.**
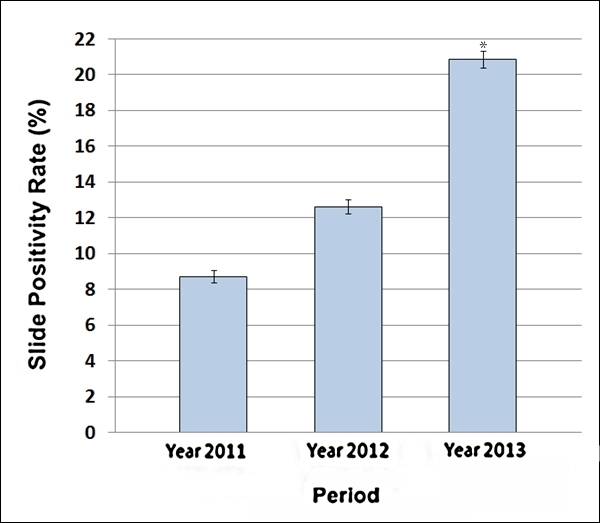

Persons exposed to the flooding in 2013 in Almanagil Locality had respectively 2.39 (95% CI: 2.27-2.51) and 1.65 times the risk of having been infected with malaria parasites compared to persons who were not exposed to flooding in 2011 and 2012 (P < 0.0001). The SPR of both age groups (< 5 years and > 5 years) increased in the 12­-week period of 2013 (P < 0.0001), particularly in the under­-5-­year age group, in comparison to the corresponding period in the non-­flooding years. SPR for the above­-5-­year age group increased from 7.79% in 2011 (95% CI: 7.41-8.18%) and 12.25% in 2012 (95% CI: 11.83-12.69%) to 19.94% in 2013 (95% CI: 19.44-20.46%). IR for the under­-5-­year age group increased from 11.94% in 2011 (95% CI: 11.09-12.84%) and 13.86% in 2012 (95% CI: 13.04-14.71%) to 24.30% in 2013 (95% CI: 23.2-25.38%) as shown in Table 4 and Figure 5.

**Table 4: Number of positive blood smears and slide positivity rate by age groups with 95% confidence intervals recorded in the SMNSs of Almanagil Locality in weeks 36-47 of years 2011-2013.**

**Figure:**
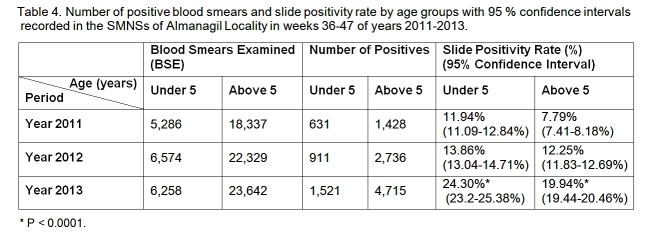

**Slide positivity rate by age groups with 95 % confidence intervals recorded in the SMNSs of Almanagil Locality in weeks 36 to 47 of years 2011 to 2013. * P < 0.0001.**
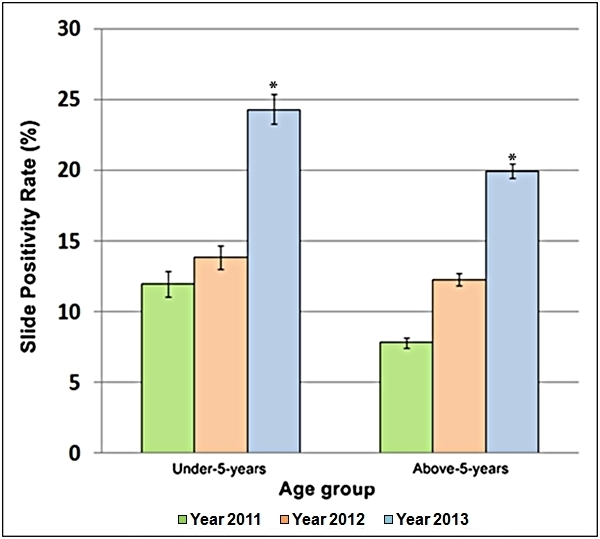

**Malaria incidence proportion in outpatient departments (OPD) of public hospitals**

A noticeable increase (P < 0.0001) in the proportion of outpatient malaria cases in public hospitals of Almanagil Locality was observed for the months September, October, November 2013 in comparison to the same period of 2011 and 2012. Hospital OPD incidence proportion in the 2013 period was 19.7% (95% CI: 19.24-­20.18%) compared to 12.85% (95% CI: 12.48-­13.23%) in 2011, and 12.16% (95% CI: 11.82-­12.51%) in 2012 (Table 5). Persons exposed to the flooding in 2013 and consulting the OPD of the public hospitals in Almanagil Locality had respectively 1.53 and 1.62 times the risk of contracting malaria compared to persons who were not exposed to flooding in 2011 and 2012.

**Table 5: Number and percentage of malaria cases out of all outpatient visits with 95 % confidence intervals in public hospitals of Almanagil Locality in the months September, October and November of the years 2011 to 2013.** 

	Number of Malaria OPD cases	Total all-cause OPD cases	Malaria cases / 100 all-cause OPD cases (95% Confidence Interval)
Year 2011 (Sep-Nov)	3,991	31,060	12.85% (12.48-13.23%)
Year 2012 (Sep-Nov)	4,211	34,624	12.16% (11.82-12.51%)
Year 2013 (Sep-Nov)	5,447	27,647	19.7% (19.24-20.18%)*
* P < 0.0001.			

A more detailed age­-specific analysis was possible as hospital data were stratified by six age groups. Analysis of the age-­specific proportion of the outpatient malaria cases revealed that the age groups responsible for the overall rise in the proportion of malaria cases in 2013 were the two under­-5-­year age groups (< 1 year and 1-­4 years). Considering < 5 years of age as one group, the proportion of outpatient malaria cases to the total number of outpatients in 2013 study period was as high as 38.5% (95% CI: 37.2-39.8%) compared to 24.3 % (95% CI: 23.0-25.6 %; P < 0.0001) in the 2011 study period and 25.2% (95% CI: 23.9-26.6%; P < 0.0001) in the 2012 study period (Table 6).

**Table 6: Percentage of malaria cases out of all outpatient visits by age group with 95 % confidence intervals in public hospitals of Almanagil Locality in the months September to November of the years 2011 to 2013.** 

	Year 2011		Year 2012		Year 2013	
Age group (in years)	Number	Proportion in % (95% CI)	Number	Proportion in % (95% CI)	Number	Proportion in % (95% CI)
Under 1	308	7.7% (6.9-8.6%)	340	8.1% (7.3-8.9%)	1,062	19.5% (18.5-20.6%)*
1 - 4	661	16.6% (15.4-17.7%)	722	17.1% (16.0-18.3%)	1,033	19.0% (17.9-20.0%)*
.						
< 5	969	24.3% (23.0-25.6%)	1,062	25.2% (23.9-26.5%)	2,095	38.5% (37.2-39.8%)*
5 -14	846	21.2% (20.0-22.5%)	898	21.3% (20.1-22.6%)	958	17.6% (16.6-18.6%)
15 - 24	833	20.9% (19.6-22.2%)	802	19.0% (17.9-20.3%)	880	16.1% (15.2-17.2%)
25 - 44	798	20.0% (18.8-21.3%)	769	18.3% (17.1-19.5%)	773	18.3% (17.1-19.5%)
45+	545	13.6% (12.6-14.8%)	680	16.1% (15.1-17.3%)	741	13.6% (12.7-14.5%)
Total	3,991	100%	4,211	100%	5,447	100%
* P < 0.0001.						

Analysis of the < 5 years of age group showed that the proportion of the hospital OPD malaria cases in the < 1 year old group more than doubled in the year of the flooding compared to the two non-flood years (Figure 6). Children < 1 year of age exposed to the flooding in 2013 had respectively 2.5 (95% CI: 2.2-2.9) and 2.4 (95% CI: 2.1-2.7) times the risk of catching malaria compared to children of < 1 year who were not exposed to flooding in 2011 and 2012 (P < 0.001).

**Comparison of the percentages of malaria cases out of all hospital outpatient visits with 95 % confidence intervals between <1 year old group and 1-4 years old age group during September to November in years 2011 to 2013. * P < 0.001.**
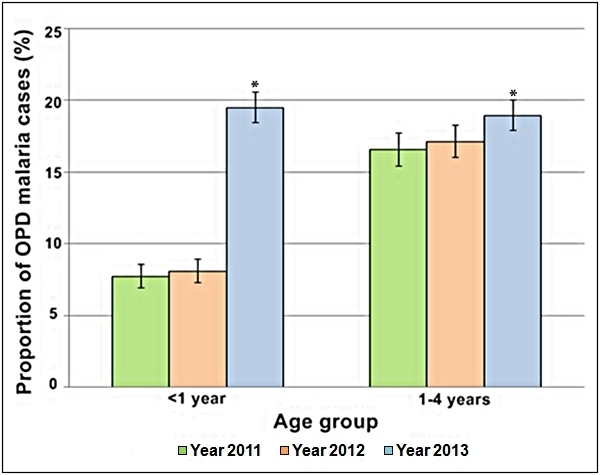

**Malaria incidence proportion of severe malaria cases in public hospitals**

The proportion of admitted malaria cases to the total hospital admissions in the 8 public hospitals of Almanagil Locality increased to 22.5 % (95% CI: 21.5-23.6%) in the study period of 2013 compared to 19.8 % (95% CI: 18.6-21.0%; P = 0.001) in 2011 and 18.4 % (95% CI: 17.4-19.5%; P < 0.0001) in 2012 as shown in Table 7.

**Table 7: Inpatient malaria cases by number and percentage of total public hospital admissions with 95 % confidence intervals in Almanagil Locality during September to November in years 2011 to 2013.** 

Period	Number of malaria admissions	Total hospital admissions	Proportion in % (95% confidence Interval)
Year 2011 (Sep-Nov)	832	4,198	19.82% (18.64-21.05%)
Year 2012 (Sep-Nov)	984	5,335	18.44% (17.43-19.51%)
Year 2013 (Sep-Nov)	1,307	5,805	22.51% (21.46-23.61%)*
* P < 0.0001.			

Analysis of age-specific proportion of the inpatient malaria cases (Table 8) showed that the increase in the proportion of severe malaria cases was due to a higher proportion of children < 5 years of age (P = 0.05) and especially to a higher proportion of children < 1 year of age (P = 0.01).

**Table 8: Percentage of malaria cases out of all hospital admissions by age group with 95 % confidence interval of malaria in public hospitals of Almanagil Locality in the months September to November of the years 2011 to 2013.** 

	Year 2011		Year 2012		Year 2013	
Age group (in years)	Number	Proportion in % (95% CI)	Number	Proportion in % (95% CI)	Number	Proportion in % (95% CI)
Under 1	101	12.1% (10.0-14.6%)	119	12.1% (10.2-14.3%)	209	16.0% (14.1-18.1%)*
1 - 4	187	22.5% (19.8-25.4%)	211	21.4% (12.0-24.1%)	298	22.8% (20.6-25.1%)
.						
< 5	288	34.6% (31.5-37.9%)	330	33.5% (30.6-36.6%)	507	38.8% (36.2-41.5%)#
5 -14	131	15.7% (13.4-18.4%)	162	16.5% (14.3-18.9%)	216	16.5% (14.6-18.6%)
15 - 24	129	15.5% (13.2-18.1%)	128	13.0% (11.0-15.2%)	153	11.7% (10.1-13.6%)
25 - 44	145	17.4% (15.0-20.2%)	181	18.4% (16.1-20.9%)	209	16.0% (14.1-18.1%)
45+	139	16.7% (14.3-19.5%)	183	18.6% (16.3-21.1%)	222	17.0% (15.0-19.1%)
Total	832	100%	984	100%	1,307	100%
* P = 0.01, # P = 0.05						

## Discussion

According to the Malaria Programme Review 2001-2012 report of Sudan’s NMCP, the national malaria incidence rate decreased from 38/100,000/day in 2000 to 9.9/100,000/day in 2011, based on both laboratory-confirmed and clinically diagnosed malaria cases reported by all outpatient departments 36 . Our study in Almanagil Locality revealed a malaria incidence rate in the non-flood years 2011 and 2012 of respectively 6.1/100,000/day and 6.48/100,000/day based only on confirmed malaria cases reported by the SMNSs (outpatient department of 4 health centres and 9 hospitals).This lower malaria incidence rate may be caused by the fact that only laboratory confirmed cases are included in the study.

Sudan is frequently exposed to flash floods due to torrential rainfall or by overflow of the river Nile during the rainy season 45^, ^46^, ^47^, ^48 . Floods may play a major role in the emergence of malaria epidemics 2^, ^7^, ^12^, ^16 . Surveys showed that floods in endemic areas in Sudan were often associated with malaria outbreaks over and above the annual increase of malaria cases normally expected in the rainy season 36^, ^45^, ^46^, ^47^, ^48 . Comprehending the interrelationship between malaria and flooding is essential for predicting epidemics, adequate preventive measures and appropriate response in order to minimize the morbidity of the affected populations 10^, ^11^, ^15^, ^49 . Malaria epidemics attributed to flooding depend on a number of factors such as the malaria endemicity and topography of the affected area, the severity of the flooding (damage to private houses and public infrastructure), the ecological change caused by the flood (propagation of malaria infected mosquitoes), displacement and vulnerability of the affected population and level and accessibility of healthcare services before and after the flood 36^, ^47^, ^50^, ^51 .

The main sources of information on malaria incidence are disease surveillance systems and health information systems operated by ministries of health. In acute emergencies such as floods, surveillance is generally based on data collected by healthcare workers and reported at regular interval through health centres and hospitals and should be representative of the entire disaster area 49 . It provides early warning of an epidemic and trends at geographical and temporal levels 49 . The study used two sources of information to collect the malaria data: a sentinel-based (SMNSs) surveillance method advocated by the NMCP and the routine health information system of the Gezira State Ministry of Health. The SMNSs of the Almanagil Locality were selected to gather information representative of the entire locality based on a number of criteria, including the coverage of hospitals and health centres and geographical areas of earlier epidemics and natural disasters 52 . The malaria incidence in this study was measured by passive case detection based on healthcare facility-based data. This assumes completeness of reporting, accurate laboratory confirmation of all malaria cases and all patients present to healthcare facilities 53^, ^54 . In order to minimize the impact of incomplete reporting, changes in healthcare utilization and errors in laboratory confirmation of malaria cases, it is recommended to focus on confirmed malaria cases, to assess trends in slide or test positivity rate (SPR/TPR) and to determine the proportion of cases that malaria make up out of all cause outpatient visits and hospital admissions 53^, ^54 . For surveillance purposes in a well-defined cohort (SMNSs), the SPR/TPR can provide a rapid and inexpensive method of estimating temporal changes in malaria incidence after a flood 27^, ^49^, ^53 . They are less affected by change in reporting rates, diagnostic practices and healthcare facility utilization rate and consider only laboratory confirmed cases of malaria 27^, ^54 . Like SPR/TPR, the proportions of outpatient and inpatient malaria cases are less sensitive to changes in reporting rates and healthcare facility utilization rate and they can reflect the burden that malaria places on the healthcare system after the flood 49^, ^54 . Change in the proportions of outpatient and inpatient malaria cases as well as SPR/TPR can reflect relative change in the malaria incidence over time, they however cannot estimate the actual incidence of malaria in a target population at national or regional level 54^, ^55 . Identifying flood-related changes in malaria incidence needs the access to reliable baseline data. An effective surveillance system is essential for providing the baseline data in order to identify flood-related changes in malaria incidence 56 .The malaria morbidity indicators in the study were extracted from surveillance and routine health information systems of the Gezira State Ministry of Health. As the analysis has been confined to SMNSs that recorded and reported consistently over time, the size of the catchment population and coverage of service utilization were stable over the evaluation period, as only laboratory-confirmed malaria cases were included in the study and no shortage in supply of microscopy supplies or RDTs in the SMNSs were observed 36 , our health facility-based data may provide a reliable indication of the malaria incidence rates over time 53^, ^54 .

The 2013 flooding in Almanagil Locality was associated with a malaria epidemic resulting in a marked and concurrent increase in the malaria incidence rate, SPR and proportion of outpatient visits and admissions in public healthcare facilities in comparison with the same period in the two previous years without floods. Age group analysis demonstrated that young children (< 5 years) are at the greatest risk from malaria in the post-flooding period because of an immature immune system and possibly under- or malnutrition, consistent with previous published reports 16^, ^57^, ^58^, ^59^, ^60^, ^61^, ^62 . In this study, the age-stratified hospital data showed that the post-flood increase in the proportion of uncomplicated and severe malaria cases in young children is mainly due to a large increase in the under-1-year age group.

Malaria epidemics following flooding are a well-known phenomenon in endemic areas worldwide 19^, ^29^, ^49^, ^50^, ^51 . Most studies attribute the increase of malaria incidence to rainfall and floods resulting in the formation of new breeding sites for the *Anopheles* mosquitoes and in providing favourable conditions for the mosquito development and survival, particularly high humidity 9^, ^10^, ^11^, ^19^, ^20^, ^21 . This is what most probably occurred in Almanagil Locality after the 2013 flood, given the presence of several factors that favour development of standing water pools and subsequent rise in the mosquito population.

Besides changes in recording completeness, healthcare utilization and diagnostic accuracy, other potential contextual factors that can confound the true malaria incidence rate include IRS, ITNs, Intermittent Presumptive Treatment of Pregnant Women (IPTp), and case management coverage 53 . IRS was implemented annually since 2000 in Gezira State. According to the NMCP, IRS in the irrigation schemes in Gezira State was consistent with the national standard method of implementation. The percentage of households covered in IRS campaigns was 97.7 % and the percentage of population protected was 98.9 % in Almanagil Locality in 2012 36 . According to the malaria indicators survey (MIS) 2009 and 2012, the percentage of households in Gezira State with at least one ITN decreased from 48 % in 2009 to 34.3 % in 2012 and the percentage of household members who slept under ITN decreased from 13.7 % in 2009 to 5 % in 2012 36. Given that the NMCP started a distribution of ITNs for target risk groups in Gezira State in 2012, it is unlikely that the use of ITNs would decrease further in 2013 36 . Since 2010 the IPTp was no longer in the national strategy and was limited to effective case management and prevention through ITNs 36 . Malaria case management guidelines and coverage in the public health facilities were unchanged over the observation period 36 . In summary, since no major changes in the possible confounding factors occurred, this study provides a strong evidence that flooding can lead to a significant increase in malaria incidence based on malaria data collected in public healthcare facilities.

## Limitations

The estimation of malaria morbidity based on healthcare facility-related data may be too low or too high due to the potential weakness of the quality of data used to measure the malaria incidence, and hence should be analysed and interpreted with caution 53^, ^54 . Possible causes include: reporting completeness, accuracy of confirmed malaria cases by microscopy or RDTs, asymptomatic and subpatent malaria infections, the extent to which patients seek treatment in public and private healthcare services or are treated at home 53^, ^54^, ^63 . In order to minimize the influence of these sources of error and bias we used the strategy recommended by the World Health Organization: focusing on confirmed malaria cases, monitoring trends in SPR/TPR (microscopic or RDTs) and monitoring malaria outpatient visits and hospital admissions. Accuracy and completeness of data could not be fully verified as retrospective data were used. The surveillance sentinel sites of the public health centres and hospitals reported consistently over time. However, a spot check of reporting completeness of Almanagil Locality health centres not included in the SMNSs showed that data were not consistently reported to the Gezira State Ministry of Health. Although the private health sector is limited in Almanagil Locality, data from the private health sector were not available during the study period. This could lead to an underestimation of malaria incidence.

## Conclusion

Overall, these results suggest that flooding following heavy precipitation has great potential to increase malaria burden in the affected population. It appears that the increase in malaria transmission occurs in the recovery phase of the flood disaster after a lag period of approximately 4 to 8 weeks. This initial delay between the flood and the post-flood malaria outbreak may provide the opportunity for vector control measures (IRS, ITNs/ Long-Lasting Insecticidal Nets [LLINs], larvicidal programmes) together with early case detection and management to mitigate the post-flood epidemic.

## Corresponding Author

Yasir E A Elsanousi, MBBCh, DTM&H, MeHM, EMDM. Email: yasir3@yahoo.com

## Competing Interest Statement

The authors have declared that no competing interests exist.

## Data Availability Statement

All relevant data are within the manuscript and the public repository Figshare at https://figshare.com/articles/S1_Dataset_rar/5458135. The DOI is: 10.6084/m9.figshare.5458135. For more information, please contact the corresponding author: Yasir E A Elsanousi, yasir3@yahoo.com.

## List of abbreviations

BSE, Blood Smears Examined

CI, Confidence Interval

FMOH, Sudanese Federal Ministry of Health

IPTp, Intermittent Presumptive Treatment of Pregnant Women

IR, Incidence Rate

IRS, Indoor-household Residual Spraying

ITNs, Insecticide Treated Nets

LLINs, Long-Lasting Insecticidal Nets

MIS, Malaria Indicators Survey

MOH, Ministry of Health

NMCP, National Malaria Control Program

OPD, Outpatient Department

RDT, Rapid Diagnostic Test

SPR, Slide Positivity Rate

SMNS, Sentinel Malaria Notification Site

TPR, Test Positivity Rate

UNDP, United Nations Development Program

WHO, World Health Organization
